# Improving the accuracy of reporting Ki-67 IHC by using an AI tool

**DOI:** 10.1016/j.heliyon.2024.e40193

**Published:** 2024-11-06

**Authors:** Sahil Ajit Saraf, Aahan Singh, Wai Po Kevin Teng, Sencer Karakaya, M. Logaswari, Kaveh Taghipour, Rajasa Jialdasani, Li Yan Khor, Kiat Hon Lim, Sathiyamoorthy Selvarajan, Vani Ravikumar, Md Ali Osama, Priti Chatterjee, Santosh KV

**Affiliations:** a Qritive Pte. Ltd, Medical Dept, Singapore, 139951, Singapore; b Qritive Pte. Ltd, Artificial Intelligence Unit, Singapore, 139951, Singapore; c Anatomical Pathology, Singapore General Hospital, Singapore, 169856, Singapore; d Duke NUS Medical School, Singapore, 169857, Singapore; e Dept of Histopathology, RV Metropolis Diagnostic & Healthcare Center Pvt Ltd, Bengaluru, Karnataka, 560003, India; f Department of Pathology, Lady Hardinge Medical College, New Delhi, 110001, India

**Keywords:** Artificial intelligence, Machine learning, Ki-67, Sarcoma, Inter-observer variability, Concordance, Immunohistochemistry, Nuclear stain, Prognosis

## Abstract

Ki-67 proliferative index (PI) scoring is measured by estimating the proportion of the number of active cell nuclei in hotspot regions within immunohistochemical (IHC) stained slides. It provides valuable information about the rate of proliferation in a tumour. Manual scoring of Ki-67 PI is laborious, time-consuming and often the victim of interobserver variability between pathologists. This motivated us to develop an AI-based method to automate Ki-67 PI scoring with the aim to improve the concordance of pathologists' inter-observability through aided diagnosis. We sourced 88 sequential cases of sarcomas for our study. We applied watershed algorithm to perform nuclear segmentation on 440 regions of interest (ROI). A study was conducted where three pathologists scored the Ki-67 PI on the ROIs with and without AI-assistance. Our study demonstrated great concordance between the pathologists scoring with AI-assistance. After AI assistance, inter-pathologist discordance was significantly reduced by 82.1 %

## Graphical abstract

**Figure:**
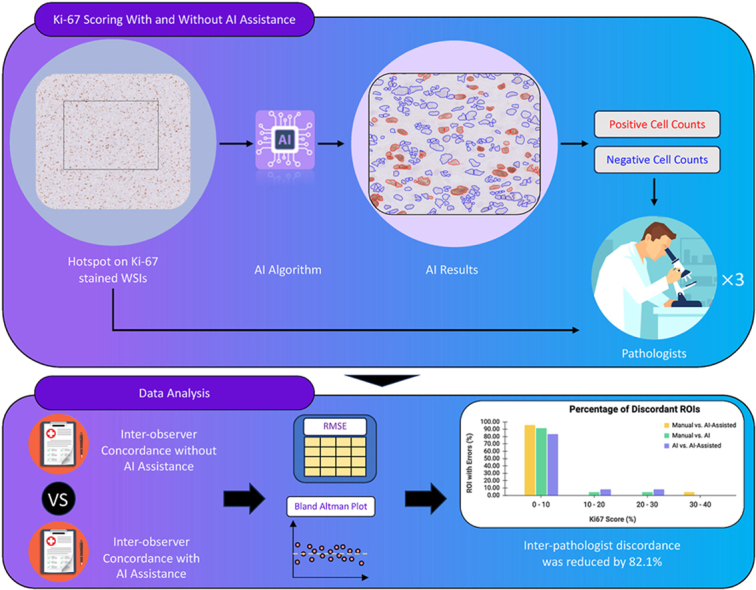

## 1 Introduction

Sarcomas are cancers of mesenchymal tissues that affect millions of lives globally, that have proven particularly complex to categorise due to the numerous entities that are encompassed, each with its own morphology and ancillary features. The annual incidence of soft tissue sarcoma is about 50 per 1 million population [1]. Grading of these tumours is performed using the French federation of cancer centers sarcoma group (FNCLCC) grading system that provides an association of tumour grade with metastatic risk, thereby providing essential prognostic details and treatment-determining information. The FNCLCC grading system comprises the three categories of differentiation, necrosis and mitotic rate [2].

While the mitotic rate remains a part of the grading system for sarcomas, the proliferative index (PI), as measured by Ki-67 monoclonal antibody expression (by immunohistochemistry), is also being used as a biomarker to evaluate tumour cell progression and predicting therapeutic responses [3,4,5]. The PI, estimated by Ki-67 expression, reflects the degree of active cell proliferation and is determined by calculating the ratio of the number of stained nuclei and the total number of nuclei. However, inter-observer variation, observer fatigue and time constraints are factors influencing the evaluation of immunohistochemically stained slides, where accurate assessment is essential to the diagnostic process [3,6].

With conventional microscopic examination, the slides of the sarcoma are screened for hotspots areas with the highest Ki-67 PI and the proportion of positively staining cells are manually counted to give the PI [7,8]. The disadvantages include a low throughput processing rate, pathologist bias and a lack of reproducibility among observers [8]. We aim to address this issue by using artificial intelligence (AI) based tools leveraging traditional computer vision methods to analyse scanned whole slide images (WSI) of Ki-67 immunohistochemical stained slides. The resultant AI-generated Ki-67 PI was used to reduce inter-observer variation between pathologists to achieve greater concordance.

## 2 Materials and methods

The individual parameters and thresholds used in this algorithm were determined experimentally and fine-tuned manually. A qualified pathologist drew region of interests (ROIs) using the annotation software available in the image management system (Qritive Pantheon). The ROIs were selected by the pathologist to include area with tumour. The model was calibrated using 15 ROIs which were separate from the ROIs used for the validation. The algorithm identified nuclei in a field of view and categorised them as positive or negative based on the intensity of the IHC staining. The AI algorithm used traditional computer vision methods to segment and classify individual cells in each region of interest (ROI). The input images consisted of two channels, Haematoxylin and diaminobenzidine (DAB).

In our work, we adopted a watershed algorithm for nuclear segmentation. A watershed algorithm is a region-based method that leverages image morphology to segregate an image into multiple segments [9]. An intensity thresholding method is applied along the Haematoxylin channel of the ROI to differentiate the background from the foreground, where pixel values greater than the threshold are considered foreground, while pixel values below the threshold are considered background. Following this, a distance transformation step is implemented to calculate the Euclidean distance between non-zero pixel and the nearest zero pixel in the ROI. The output of the transformation is then normalized, yielding a distance map that contains information about the peaks in the ROI. These peaks will serve as seeds for the watershed algorithm to perform nuclear segmentation. Lastly, the intensity of staining from the DAB channel for each of these detected nuclei was used to determine whether the corresponding cell was positive or negative. The final percentage of positive cells was used to determine the final Ki-67 PI for the ROI that the AI algorithm was run on.

**Fig. 1:**
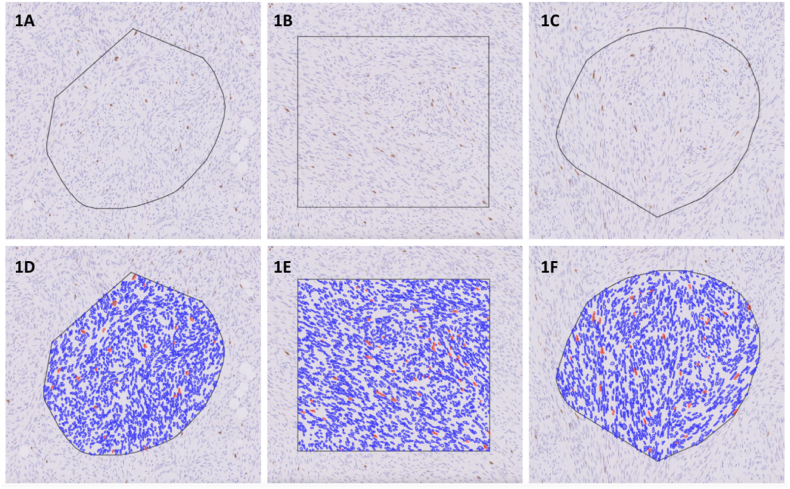
**Manually drawn ROIs with AI outputs.** 1A, 1B, 1C are ROIs without AI outputs drawn by our experts. 1D, 1E, 1F are ROIs with AI outputs, with red annotations indicating positive (DAB) cells and blue annotations indicating negative (haematoxylin) cells.

A total of 88 sequential cases of sarcomas were sourced from archival material from Singapore General Hospital for the study. Deidentified WSI of needle biopsies and resection specimens that had been subjected to Ki-67 immunohistochemical stain were used. The histopathology diagnoses were not taken into consideration and the original Ki-67 scores were not retrieved. Each slide had five random ROIs drawn by in-house annotators, as shown in Fig. 1A, B and 1C leading to a total of 440 ROIs for analysis (areas ranging from 0.0203 mm^2^ to 3.1473 mm^2^). The ROIs were then evaluated by 3 pathologists independently. There were two rounds (phases) of interpretations and staining of any intensity was considered positive. In the first round (phase 1) each pathologist scored the Ki-67 PI at 5–10 % intervals by visual assessment (as a percentage of Ki-67 positive nuclei out of the total nuclei within the ROI). The first round was done without any AI assistance. Following the first round, a washout period of 2 weeks was observed before starting the second round. In the second round (phase 2) the pathologists assessed the Ki-67 PI on all the 440 ROIs in the same manner but this time with assistance from the AI output. In the second round, the estimated Ki-67 PI of each ROI and the cell segmentation outputs that were derived from the AI were made available to the reporting doctors. The discordance between the pathologist’s initial score and the final score after viewing the AI outputs was calculated for each pathologist.

## 3 Results

Fig. 1 depict the samples from our dataset of Ki-67 stained slides. The black polygons in Fig. 1A and C as well as the black rectangle in Fig. 1B are manually drawn ROIs. Fig. 1A, B and 1C are snapshots without the AI output, while Fig. 1D, E and 1F show the AI output, the nuclei staining positive for Ki-67 (DAB) are identified by red borders and the negatively stained nuclei (haematoxylin) are identified by blue borders. Fig. 1D shows 98.6 % negative cells and 1.4 % positive cells within the ROI, Fig. 1E shows 97.5 % negative cells and 2.5 % positive cells within the ROI, and Fig. 1F shows 97.5 % negative cells and 2.5 % positive cells within the ROI. In other words, the PIs for Fig. 1D, E and 1F were 1.4 %, 2.5 % and 2.5 % respectively. These precise outputs were generated by the AI once the instruction to run the AI was given to the system.

1. Inter-observer agreement

To quantify the degree of difference in Ki-67 evaluation between each respective pathologist, with and without knowledge of the AI output, the root mean squared error (RMSE) was performed and results tabulated as shown in Table 1. The RMSE between pathologist 1 (P1) and pathologist 2 (P2), without knowledge of AI’s input was 18.10, that between P2 and pathologist 3 (P3) was 12.12 and that between P1 and P3 was 12.01, with an average discordance of 14.08. In contrast, the RMSE calculated between pathologists after each pathologist was provided the AIs’ input, was 2.53 between P1 and P2, 2.93 between P2 and P3 and 2.10 between P1 and P3, with an average discordance of 2.52 (Table 1), demonstrating that after AI assistance, inter-pathologist discordance was significantly reduced by 82.1 %.

**Table 1:** Inter-observer RMSE correlation matrix.

Root Mean Squared Error (RMSE)							
	**Pl**	**P2**	**P3**	**Al**	**P1-AI**	**P2-AI**	**P3-AI**
**Pl**	0.00	18.10	12.01	17.13	17.13	17.50	16.89
**P2**		0.00	12.12	5.07	4.90	4.00	4.99
**P3**			0.00	11.70	11.54	11.90	11.43
**Al**				0.00	2.18	2.69	3.05
**P1-AI**					0.00	2.53	2.10
**P2-Al**						0.00	2.93
**P3-Al**							0.00

This table shows the discordance between the pathologists after AI assistance. P1, P2, P3 are pathologists’ scores before AI assistance. P1-AI, P2-AI, P3-AI are pathologists’ scores after AI assistance. AI denotes the Ki-67 scores given by the AI system. Each cell represents discordance measured by root mean square error (RMSE) of pairs of variables.

**Fig. 2:**
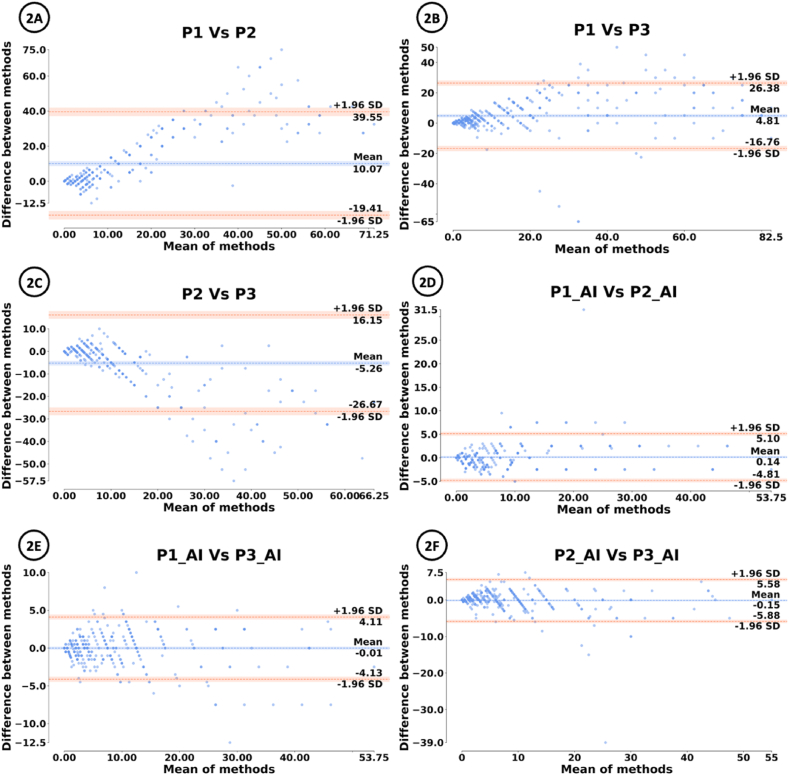
Bland-Altman plots for inter-observability agreement.

In this study, we employed Bland-Altman plot to measure the inter and intra-observability concordance of Ki-67 evaluation between pathologist, AI, and pathologist with AI-assistance. Bland-Altman plot is an analysis that measures the agreement between two methods. In a Bland-Altman plot, the differences between the two methods are plotted against the average of the two methods. The mean difference between the two methods is plotted as a horizontal line on the graph. This line (dashed blue in Fig. 2) indicates the average discrepancy between the two methods. Two horizontal lines (dashed orange in Fig. 2) are drawn above and below the mean difference line representing the limits of agreement. The limits of agreement are calculated as the mean difference ±1.96 times the standard deviation of the differences. The confidence interval provides a range of values which the limits of agreement are likely to lie with a certain level of confidence. A wider confidence interval indicates greater uncertainty in estimating the limits of agreement, whereas a narrower confidence interval suggests more precise estimates. Statistical confidence interval has an upper threshold and a lower threshold. In this study, the level of confidence was set to 95 %. This indicates the proportion of the confidence interval that would capture the true population parameter. The trend of increased inter-pathologist concordance after AI-assistance is evident through the analysis of agreements between two methods as shown in the Bland-Altman plot (Fig. 2). A low discordance was observed for inter-pathologist agreement with the knowledge of AI’s input (Fig. 2D, E, 2F), as opposed to inter-pathologist agreement prior to AI’s input (Fig. 2A, B, 2C).

Fig. 2A shows that the overall mean difference between P1 and P2 is 10.07 with 95 % confidence interval ranging from −19.41 to 39.55. Fig. 2B shows that the overall mean difference between P1 and P3 is 4.81 with 95 % confidence interval ranging from −16.76 to 26.38. Fig. 2C shows that the overall mean difference between P2 and P3 is −5.26 with 95 % confidence interval ranging from −26.67 to 16.15. Fig. 2D shows that the overall mean difference between P1_AI and P2_AI is 0.14 with 95 % confidence interval ranging from −4.81 to 5.10. Fig. 2E shows that the overall mean difference between P1_AI and P3_AI is −0.01 with 95 % confidence interval ranging from −4.13 to 4.11. Fig. 2F shows that the overall mean difference between P2_AI and P3_AI is −0.15 with 95 % confidence interval ranging from −5.88 to 5.58.

Fig. 2A, B and 2C show Bland-Altman plot of inter-observer agreement between pathologists (P1, P2 and P3) without the AI's input. Fig. 2D, E and 2F show Bland-Altman plot of inter-observer agreement between pathologist with the AI's input (P1_AI, P2_AI, P3_AI).

2. Intra-observer agreement

The concordance between the three pathologists was improved after they had knowledge of the AI’s output, though the degree to which this occurred was variable. In the case of P2, the difference between the manual and AI-assisted results was within 7.5 %, while for P1 and P3, the differences with the AI-assisted results were 30.0 % and 57.5 % respectively. This demonstrated the ability of the AI to reduce intra-observer variability.

**Fig. 3:**
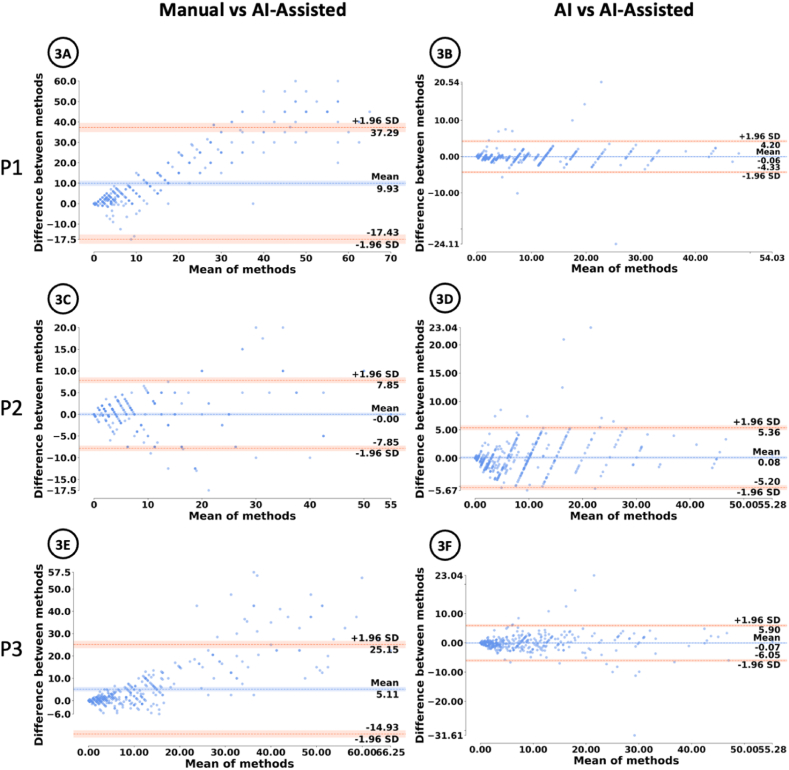
Bland-Altman plots for intra-observability agreement.

Fig. 3A, C and 3E show Bland-Altman plot of intra-observability between manual and AI-assisted scoring for each pathologist (P1, P2 and P3). Fig. 3B, D and 3F show Bland-Altman plot of intra-observability between solely AI and AI-assisted scoring for each pathologist (P1, P2 and P3).

**Fig. 4:**
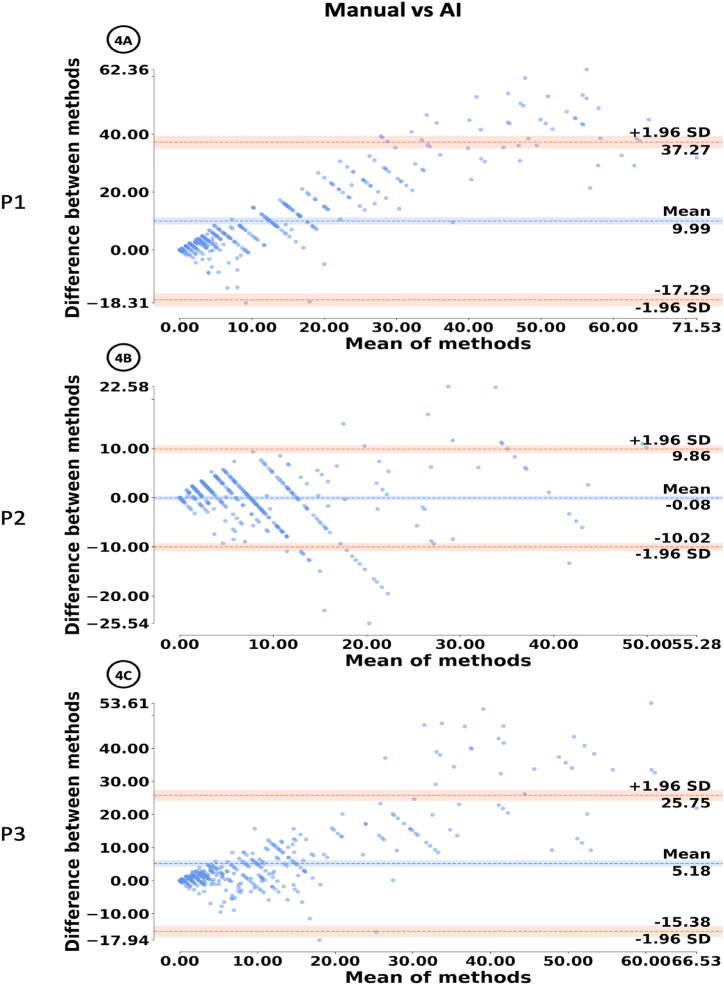
Bland-Altman plots for intra-observability agreement between manual and AI-assisted scoring.

Fig. 4A, B and 4C show Bland-Altman plot for each pathologist (P1, P2 and P3).

Fig. 3A, C and 3E compare the manual scoring and the AI-Assisted scoring method. As seen from the cluster of points near 0 for each pathologist, the graph indicates that the majority of the differences in Ki-67 scoring lie between 0 % and 10 % (Fig. 3A), −5% and 5 % (Fig. 3C), −5% and 10 % (Fig. 3E). Fig. 4A, B and 4C compare the manual scoring method and the Ki-67 score predicted by the AI. Fig. 3B, D and 3F show the comparison between the Ki-67 score produced by the AI and the AI-Assisted score given by the pathologist. For most ROIs, almost all of the points are within the 95 % confidence interval, indicating that the pathologists aligned with the scores generated by the AI.

### 3.1 Agreement with AI

From Table 2, the metrics calculated show that the pathologists agreed with 93.5 % of the ROIs scored by the AI (number of ROIs accepted/total number of ROIs in the study). In the instances where a pathologist’s assessment diverged from that of the AI-generated results (Fig. 2), the primary reason for the discordance was attributed to the detection of minor foci of background debris falsely detected by the AI as positive.

**Table 2:** Concordance between three pathologists (P1, P2 and P3) with respect to AI outputs.

	P1	P2	P3	Mean
**Concordance ROIs**	416	415	404	411.7
**Agreement Rate (%)**	94.6	94.3	91.8	93.5

Concordance ROIs indicate the number of ROIs with AI outputs accepted by pathologists out of the 440 ROIs.

**Fig. 5:**
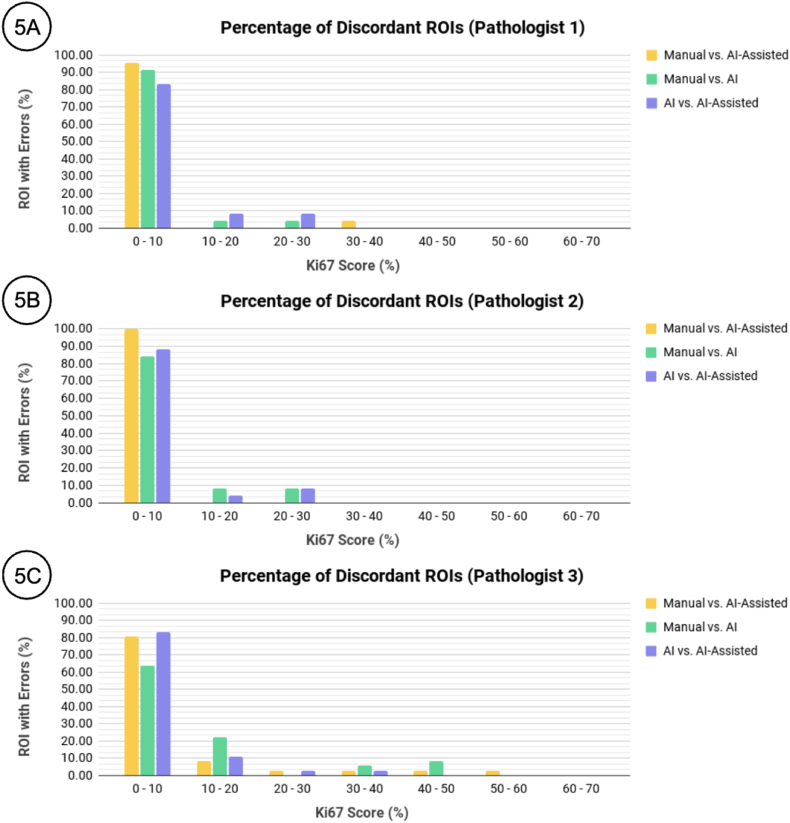
Distribution of errors while scoring Ki-67 on the selected ROIs.

Fig. 5A, B, and 5C show that most of the errors occurred on ROIs with low scores between 0 and 10 % PI. Subsequent intervals of 10 % PI showed fewer errors. The maximum discordance noted was where pathologist 3 manually scored an ROI as 65.0 % PI, and then significantly reduced the score to 7.5 % PI after viewing the AI output. This amounts to an absolute error of 57.5 % which falls into the 50–60 % PI the scoring bracket shown in Fig. 5C. Fig. 5 also shows a distribution shift, most notably in the scoring bracket of 0–10 % PI, when comparing the ROI with error counts of manual scoring with AI-assisted (yellow bar) and without AI-assisted (green bar).

## 4 Discussion

In anatomical pathology, microscopic examination of the tissue remains the gold standard for achieving an accurate diagnosis and to assess specific parameters for prognosis. However, inter-observer variability in interpretation can lead to inaccurate results [7,8]. To attain a greater degree of accuracy, AI has been relied upon in several areas of pathology [9,10].

Inter-observer variability in the assessment of Ki-67 index has been extensively studied in breast cancers. These studies assessed variability across different ranges, hot spot scoring versus average scoring and scoring in predetermined areas as against the average score [[11], [12], [13]]. The use of AI can reduce discordance and lead to improved agreement among pathologists assessing Ki-67. Studies on breast cancers have shown that digital evaluations of Ki-67 index are better than visual estimations [14,15]. One study recruited 90 international pathologists to assess Ki-67 PI of 10 breast cancer tissue micro arrays (TMAs) with and without AI. Using the AI resulted in a significant decrease in PI error with the P-score less than 0.001. The authors also found an overall 11.9 % reduction in turnaround time. 84 % of the pathologists found the AI reliable in the study ^17, 18.^ A second study was conducted on 300 invasive breast cancer, with varying Ki-67 PIs. 9 pathologists accessed these cases. This study showed that with the use of AI intra-observer agreement was very good with an intra-group correlation coefficient (ICC) more than 0.9, showing that AI has a satisfactory inter-observer repeatability [16]. A third study use 7 pathologists 72 breast cancer specimens and found similar levels of inter-observer agreement and also reported that it took less time to score the slides with AI (229 s per slide) than (306 s per slide) without [17]. As the AI outputs are not influenced by manual evaluation, they remain an independent and objective means of measurement, and are a useful adjunct in supporting pathologists’ assessments.

Rarely, our algorithm would overcall highly pleomorphic enlarged cells, leading to a falsely increased Ki-67 PI within an ROI. This was mitigated by calibrating the algorithm during the development process. As such, manual verification of the Ki-67 PI AI output by the pathologist is still essential. Though AI-assistance has been useful in achieving concordance amongst pathologists, we acknowledge that the process of manually demarcating ROIs continues to be influenced by each individual pathologist, potentially increasing inter-observer variation in this process.

The Ki-67 PI has been used in previous studies in place of the mitotic count advocated under the FNCLCC system and has shown better reproducibility between pathologists and correlation with the histological grade of soft tissue sarcomas [18,19].

In WSI analysis, the manual detection of highly proliferative areas along with calculation of proliferative indices is labour intensive and time consuming. The present study utilises AI for calculating Ki-67 scoring in soft tissue sarcomas. WSI-based AI software is an effective method for Ki-67 evaluation and has a higher consistency and accuracy than visual evaluation.

With regards to the limitations in the study, we found that the use of manually drawn ROIs was necessary because currently the AI algorithm is unable to differentiate between tumour areas and background normal tissue. The AI is also unable to separate tumour from background inflammation. Subsequent version of this AI will include these capabilities. The data used in this study was sourced from a single institute (Singapore General Hospital) and we recommend that further studies source data from at least two to three institutes as the practice of reporting and image qualities may differ.

Most studies with AI system that quantified Ki-67 are done on breast cancer and other tissue type are underrepresenting. We did this study on sarcomas because we wanted to test the system on the spectrum of cases some of which showed bizarre morphology. This was useful because initially we found that the AI system was unable to detect large nuclei as a single nucleus and tended to divide these nuclei into many. Subsequently, we calibrated the system to resolve this discrepancy. We have also used Bland-Altman plots to display the inter (between the 3 pathologists) and intra-observer variability (individual pathologist with and without the use of AI). These Bland-Altman plots enable the reader to visualize very fine details with regards to inter and intra-observer variability.

## 5 Conclusion

In conclusion, AI-assisted evaluations combine the expertise of the pathologist in selection of ROI and the strength of AI to accurately identify and count tumour cells to achieve greater concordance and reproducibility to ensure accurate prognostic and treatment outcomes.

## CRediT authorship contribution statement

**Sahil Ajit Saraf:** Writing – review & editing, Writing – original draft, Visualization, Validation, Supervision, Project administration, Methodology, Investigation, Formal analysis, Data curation, Conceptualization. **Aahan Singh:** Validation, Software, Investigation, Formal analysis, Data curation. **Wai Po Kevin Teng:** Writing – review & editing, Writing – original draft, Supervision, Methodology, Investigation, Data curation, Conceptualization. **Sencer Karakaya:** Writing – review & editing, Writing – original draft, Validation, Software, Methodology, Investigation, Formal analysis, Data curation. **M. Logaswari:** Validation, Investigation, Formal analysis, Data curation. **Kaveh Taghipour:** Validation, Supervision, Methodology, Funding acquisition, Formal analysis, Conceptualization. **Rajasa Jialdasani:** Validation, Investigation. **Li Yan Khor:** Writing – review & editing, Writing – original draft, Validation, Methodology, Investigation, Formal analysis, Data curation, Conceptualization. **Kiat Hon Lim:** Supervision, Resources, Investigation, Data curation, Conceptualization. **Sathiyamoorthy Selvarajan:** Resources, Methodology, Conceptualization. **Vani Ravikumar:** Validation, Investigation, Formal analysis, Conceptualization. **Md Ali Osama:** Writing – review & editing, Writing – original draft, Validation, Investigation, Formal analysis. **Priti Chatterjee:** Writing – review & editing, Writing – original draft, Validation, Investigation, Formal analysis. **Santosh KV:** Writing – review & editing, Writing – original draft, Visualization, Validation, Methodology, Formal analysis.

## Ethics and consent

We use completely anonymised whole slide images for this research. There were no patient identifiers in the dataset that we used. The Sing health centralised institutional review board determined that since the data used in this study was deidentified, ethics waiver was granted for this study.

Ethics Approving Commitee: Sing health Centralised Institutional Review Board (CIRB)

Ethics Approval Number: 2020/2036.

Ethics Approval Date: 16^th^ January 2020.

## Disclosures

Sahil Ajit Saraf, Aahan Singh, Wai Po Kevin Teng, Sencer Karakaya, Kaveh Taghipour, and Rajasa Jialdasani are Qritive employees. The other authors have no disclosures.

## Declaration of competing interest

The authors declare the following financial interests/personal relationships which may be considered as potential competing interests: Sahil Ajit Saraf reports financial support and administrative support were provided by Qritive Pte Ltd. Sahil Ajit Saraf reports a relationship with Qritive Pte Ltd that includes: employment. Kaveh Taghipour reports a relationship with Qritive Pte Ltd that includes: employment. Rajasa Jialdasani reports a relationship with Qritive Pte Ltd that includes: employment. Wai Po Kevin Teng reports a relationship with Qritive Pte Ltd that includes: employment. Aahan Singh reports a relationship with Qritive Pte Ltd that includes: employment. Sencer Karakaya reports a relationship with Qritive Pte Ltd that includes: employment. None to declare. If there are other authors, they declare that they have no known competing financial interests or personal relationships that could have appeared to influence the work reported in this paper.
